# Improving the Wearing Rate of Mouthguards in the Youth Rugby Category Affects the Total Future Mouthguard Wearing Rate

**DOI:** 10.3390/dj8030077

**Published:** 2020-07-27

**Authors:** Kairi Hayashi, Hiroshi Churei, Gen Tanabe, Kaito Togawa, Ruman Uddin Chowdhury, Toshiaki Ueno

**Affiliations:** 1 Department of Sports Medicine/Dentistry, Graduate School of Medical and Dental Sciences, Tokyo Medical and Dental University, 1-5-45 Yushima, Bunkyo-ku, Tokyo 113-8549, Japan; k.hayashi.spmdl@tmd.ac.jp (K.H.); chu.spmd@tmd.ac.jp (H.C.); genspmd@tmd.ac.jp (G.T.); kaito321.629@gmail.com (K.T.); 2 Department of Orthodontics, Kumudini Women’s Medical College, Dhaka University, Nilkhet Road, Dhaka 1000, Bangladesh; ruman_chowdhury@yahoo.com

**Keywords:** mouthguard, wearing rate, rugby, young athletes, custom-made mouthguard

## Abstract

The aim of this study is to consider the hypothesis that wearing mouthguards (MGs) from a young age helps to shape players’ habit of continuing to wear MGs as adults. In addition, we consider the mandatory rule of wearing mouthguards in the youth category on the future wearing rates of mouthguards. Eighty-five Japanese male amateur rugby players were included in this study. We examined the relationship between the wearing rates of mouthguards when participants were high school students and as adults (in 2018) using a questionnaire. Collected data were classified into categories (age, mouthguard type, and applied mandatory rule or not), and their relationships were analyzed. The wearing rate of mouthguards in high school was significantly related to the wearing rate of mouthguards in adulthood (χ2 = 12.1, *p* < 0.01). The mandatory rule to wear mouthguards at high school was related to the wearing rates of mouthguards as adults, but not significantly (χ2 = 3.42, *p* = 0.06). Participants’ mouthguard wearing rates as adults were affected by their high school wearing rates. The mandatory rule of wearing mouthguards in the youth category may be somewhat effective for improving mouthguard wearing rates; however, it is more important to provide knowledge about their importance.

## 1. Introduction

Sports, especially collision sports, have a high risk of dental trauma [1,2,3,4,5]. Therefore, wearing mouthguards (MGs) is recommended in some sports to prevent orofacial injury, and the efficacy of MGs has been shown in many reports. Many in vitro studies have investigated the ability of MG materials to protect teeth and oral tissues. For example, Fukasawa et al. investigated the shock-absorbing capability of commercial thermoforming MG materials. They suggested that all commercially available mouthguard sheet materials have shock-absorbing capabilities (they reduce impact energy by 62%–74%, 2 mm thickness) that are high enough to protect the teeth and oral tissue [6]. In addition, many clinical studies have also suggested that MGs prevent orofacial injury. For example, Berg et al. reported a survey on the history of orofacial injury in wrestling, basketball, baseball, softball, soccer, and volleyball players in American high schools, finding that the incidence rates of orofacial injury in the group who wore MGs were less than in the group that did not [7]. Finch et al. reported a controlled randomized trial to evaluate the effectiveness of MGs in preventing orofacial injuries in Australian football players, suggesting that custom-made MGs had a significant protective effect [8]. Quarrie et al. investigated the economic effects of wearing MGs. In New Zealand, wearing MGs during rugby matches was made compulsory for all grades of players at the beginning of the 1998 season. They surveyed and compared the incidence rates of orofacial injuries between 1995 and 2003, finding the incidence rate of orofacial injury in 2003 to be 43% less than in 1995; the medical cost in New Zealand was reduced by a total of 1.87 million NZD [9].

In 2008, based on many reports on preventing orofacial injury by wearing MGs [10,11,12,13], the FDI (World Dental Federation) issued an official statement that recommended the utilization of MGs to prevent dental injury. They also stated that a custom-made MG provides the best level of protection and wearer comfort, that mouth-formed MGs are less adequate, and that stock mouth guards provide the lowest level of protection and wearer comfort [14]. In addition, the FDI stated that customized sports mouthguards should be manufactured under the supervision of a dentist in a statement on “Sports Dentistry” in 2016 [15].

Wearing MGs is mandatory in boxing, lacrosse, and other sports. In these sports, this rule is applied in all categories and throughout the world [16,17,18]. However, there are no rules requiring MGs in some sports because of such issues as the financial burden and discomfort from wearing an MG. Against such a background, in some sports, such as rugby in Japan, a mandatory rule to wear MGs has been applied restrictively only to the youth categories [19]. In Japan, the mandatory rule to wear an MG was applied to high school rugby players (U-18) during games in 2006. Wearing MGs through the youth categories is effective in protecting immature teeth and orofacial bones. In addition, wearing MGs from youth may help to shape players’ habits to continue wearing MGs in adulthood.

Many studies have examined the wearing rate of MGs in cross-sectional studies [20,21,22,23]. However, there have been no studies of changes in the MG-wearing rate for the same athletes, and no reports have verified whether the application of compulsory MGs would affect MG-wearing habits without a mandatory rule.

We hypothesize that different earlier experiences have an influence on MG wearing rates as adults. Hence, in this study, we investigate the relationship of MG wearing rates in high school and as adults via questionnaires. In addition, we analyze whether the application of a mandatory rule of MG wear that is restricted to the youth category affects players’ current MG wearing rates. The results of this study may be so useful for developing strategies to improve the wearing rates of MGs.

## 2. Materials and Methods 

### 2.1. Study Design

This survey was conducted from January to April 2018 by administering questionnaires to 85 male amateur rugby players. The results were then analyzed to investigate the relationship between wearing MGs in 2018 and in high school. This study was approved by the Ethics Committee of the Tokyo Medical and Dental University Faculty of Dentistry (D2017-064) on 19 January 2018, and the subjects gave their informed consent according to institutional guidelines.

### 2.2. Participants

A total of 85 Japanese male rugby players (20–60 years old, mean ± SD: 36.2 ± 11.7) who belonged to amateur rugby clubs that had no “team dentist” were included in the study. In Japan, top-level teams are supported by so-called “team dentists”. All of them had begun to play rugby when they were high school students. Individuals 30 years old or younger during the study term (January to April 2018) were subject to the mandatory rule of wearing MGs when they were high school students. All of them trained or played rugby at least once per week for Japanese amateur club tournaments or amateur club senior tournaments. 

### 2.3. Questionnaire

The question items were age, presence or absence of MG use in high school and in 2018, the type of MG (custom-made, mouth-formed, stock) used in high school and in 2018, and frequency of use (training, match, both) in high school and in 2018 (Table 1).

### 2.4. MG Wearing Rates Analyzed by Age Classification

We classified participants as ≤30 years old (*n* = 29; subject to mandatory MG use in high school), 31–39 years old (*n* = 27), and ≥40 years old (*n* = 29). Then, we calculated the wearing rates of MGs by age group.

### 2.5. Relationship between MG Usage of Participants Currently and in High School

We analyzed the relationship between MG wearing rates of custom-made MGs at high school and as adults.

### 2.6. Analyzing the Effect of Compulsory MG Wear at High School

To investigate the effect of compulsory MG wear in high school, we classified the participants as ≤30 years (use of MGs in high school was mandatory) and ≥31 years (not subject to such a mandatory rule) and calculated the MG wearing rates in 2018.

### 2.7. Statistical Analysis

The statistical significance of the results was analyzed using Pearson’s chi-squared test. All statistical analyses were performed with statistical software (Ekuseru–Toukei 2015, Social Survey Research Information Co., Ltd., Tokyo, Japan). A *p*-value <0.05 was considered significant.

## 3. Results

### 3.1. Data of Participants and Their Usage of MGs

The participants’ age and distribution are shown in Table 2. 

Table 3 shows the MG wearing rates of participants at the time of investigation (2018) and at high school. MG usage in 2018 was 62.4% overall, and the highest wearing rate was found in participants aged 30 years or younger (75.9%), followed by 31–39 years old (66.7%), and then, 40 years or older (44.8%). When participants were high school students, 52 participants (61.2%) used an MG and 44 participants used custom-made types, while in 2018, 53 participants (62.4%) used an MG and 44 of them used a custom-made type. No participants used stock-type MGs in 2018 or in high school.

The frequency of MG use is shown in Table 4. MGs were used by 21 participants (40.4%) in both training and matches in high school, while 33 participants (62.3%) used MGs in both training and matches in 2018. The wearing rates showed no significant difference between the past and present; however, the current frequency of use was higher than in high school except among those aged 30 years or younger. 

### 3.2. MG Wearing Rates of Participants Currently and in High School

Statistical analysis found a significance between MG wearing rates at high school and at the time of investigation in 2018 (χ2 = 12.1, *p* < 0.01, Table 5), between “custom-made” MG wearing rates of participants in 2018 and “overall” MG wearing rates in high school (χ2 = 5.12, *p* < 0.05, Table 6), and between “custom-made” MG wearing rates of participants in 2018 and in high school (χ2 = 12.8, *p* < 0.01, Table 6).

### 3.3. Analyzing the Effect of Compulsory MG Wear at High School

We classified the participants into whether they were subject to the mandatory rule in high school or not. The wearing rate in 2018 of participants subject to mandatory MG wear in high school was 75.9% versus 55.4% in the others (χ2 = 3.42, *p* = 0.06, Table 7). 

## 4. Discussion

This study investigated the MG wearing rates as adolescents at high school and as adults in Japanese male amateur rugby players. In addition, we analyzed the effect of a mandatory rule of wearing MGs that is restricted to the youth categories on differences in MG wearing rates.

Japanese male rugby players who belonged to an amateur club without a “team dentist” were chosen as participants. In Japan, awareness of MGs is increasing, and many top-level clubs have a “team dentist” who is a medical professional. They strongly recommend MGs to athletes, so, almost all top-level rugby players in Japan wear custom-made MGs. However, almost all amateur clubs have no “team dentist”. To examine practical personal awareness about MGs, we thus chose the participants in this study.

We classified participants into three groups by age: 30 years old or younger, 31–39 years old, and 40 years old or older. In Japan, rugby players 30 years old or younger in 2018 would have been subject to the mandatory rule of wearing MGs as high school students. In addition, rugby players 40 years old or older are permitted to join senior-rule rugby in Japan. We classified the participants into three groups for this reason. In the analysis of MG wearing rates by age, the wearing rate of MGs was higher in the youngest group in both 2018 and at high school, due to improvements in MG awareness and the efforts of many dentists. Numerous surveys about MG awareness have been conducted in many sports all over the world [24,25,26], suggesting that MG education, knowledge of dental trauma, and MG comfort are factors related to MG wearing rates. In Japan, many dentists have made significant efforts to promote MGs, the result of which the Japanese Ministry of Education, Culture, Sports, Science and Technology (MEXT) stated that the government and the public organizations should make an effort to improve knowledge of the effects of wearing MGs among players and coaches in the “Basic Plan of Sports (2012)” [27].

Analysis of the total wearing rates of MGs showed no change between adolescents at high school and the adults investigated in 2018; however, the frequency of use (training, match, both) in 2018 was higher than in high school except among those aged 30 years or younger. Most players who were 31 years old or older used MGs both in matches and training. However, younger players often behave in ways inconsistent with their long-term best interests because their behaviors are often based on avoiding immediate effort, cost, and time burdens [28].

The MG wearing rates of participants who wore an MG in high school were significantly higher. In addition, the custom-made MG wearing rate in 2018 also shows an association with the MG wearing rate at high school. In particular, participants who wore custom-made MGs in high school were extremely likely to use custom-made MGs in 2018 as well.

The current wearing rate of MGs in participants subject to the mandatory rule was higher than in the other group, but not significantly, suggesting that using MGs in adolescence was effective in improving the total MG wearing rates, and the mandatory rule may help improve the future wearing rate; however, only the application of the rule in itself did not improve the total wearing rate.

In other words, players who did not grasp the importance of using MGs had stopped doing so after the mandatory rule no longer applied to them. To ensure the use of MGs continuously in addition to the application of the mandatory rule of wearing an MG, we should make players use MGs of their own will. 

Quarrie investigated the effect of a mandatory MG rule for all categories of players in New Zealand [9]. However, we did not find the MG wearing rules in different countries in the literature. Of course, there has been no study of the categorized mandatory rule regarding MGs. Therefore, the data on wearing rates around ten years after the categorized mandatory rule was implemented are very valuable; however, the number of participants in this study is rather small.

## 5. Conclusions

Even though further investigations are needed, this study indicates that wearing MGs as young players is related to improved MG wearing rates at a later age. The categorized mandatory rule of wearing MGs in the youth category of rugby players may be somewhat effective in improving MG wearing rates; however, it was found to be more important to provide custom-made MGs and impart to young players the importance of wearing MGs.

## Figures and Tables

**Table 1:** Questionnaire.

No	Question
1	How old are you?
2	Have you ever used a mouthguard when you were a high school student?
3	If yes, what type of mouthguard did you use (custom-made, mouth-formed, stock)?
4	If yes, how frequently did you used a mouthguard (only match, only training, both)?
5	Do you use a mouthguard now?
6	If yes, what type of mouthguard do you use (custom-made, mouth-formed, stock)?
7	If yes, how frequently do you use a mouthguard now (only match, only training, both)?

**Table 2:** Age classification of the participants.

Group	Number	Age
≧40 y	29	41–60 (50.8 ± 5.3)
31–39 y	27	32–39 (33.1 ± 2.4)
20–30 y	29	20–29 (24.4 ± 2.8)
total	85	20–60 (36.2 ± 11.7)

**Table 3:** MG wearing rates of study participants.

	Group	Number of Participants who Do (Did) Not Wear a MG	Number of Participants Who Wear (Wore) a MG	Number of Participants Who Wear (Wore) a Custom-Made Type MG	Number of Participants Who Wear (Wore) a Mouth-Formed Type MG
In 2018	Total	32 (37.6%)	53 (62.4%)	44 (51.8%)	9 (10.6%)
	≧40 y	16 (55.2%)	13 (44.8%)	10 (34.5%)	3 (10.3)
	31–39 y	9 (33.3%)	18 (66.7%)	16 (59.3%)	2 (7.4%)
	20–30 y	7 (24.1%)	22 (75.9%)	18 (62.1%)	4 (13.8%)
In high school	Total	33 (38.8%)	52 (61.2%)	44 (51.8%)	8 (9.4%)
	≧40 y	24 (82.8%)	5 (17.2%)	3 (10.3%)	2 (6.9%)
	31–39 y	9 (33.3%)	18 (66.7%)	17 (63.0%)	1 (3.7%)
	20–30 y	0 (0%)	29 (100%)	24 (82.8%)	5 (17.2%)

**Table 4:** MG wearing frequencies.

**Group**	**Stage**	**Both**	**Only Match**	**Only Training**
Total	High school	21 (40.4%)	28 (53.8%)	3 (5.8%)
	In 2018	33 (62.3%)	20 (37.7%)	0 (0.0%)
≧40 y	High school	1 (20.0%)	2 (40.0%)	2 (40.0%)
	In 2018	7 (53.8%)	6 (46.2%)	0 (0.0%)
31–39 y	High school	5 (27.8%)	12 (66.7%)	1 (5.6%)
	In 2018	14 (77.8%)	4 (22.2%)	0 (0.0%)
20–30 y	High school	15 (51.7%)	14 (48.3%)	0 (0.0%)
	In 2018	12 (54.5%)	10 (45.5%)	0 (0.0%)

**Table 5:** MG wearing rates of participants at high school and at the investigation (2018).

	No MG Use in 2018	MG Use in 2018	*p*-Value	Chi-Square Score
No MG use when participants were high school students	20 (60.6%)	13 (39.4%)	0.0005	12.113
Participants used MG when at high school	12 (23.1%)	40 (76.9%)		

**Table 6:** Relationship between MG usage in high school and custom-made MG wearing rates.

	No Custom-Made MG Use in 2018	Custom-Made MG Use in 2018	*p*-Value	Chi-Square Score
No MG use at high school	21 (63.6%)	12 (36.4%)	0.0236	5.1243
Paticipants used MG at high school	20 (38.5%)	32 (61.5%)		
No custom-made MG use at high school	28 (68.3%)	13 (31.7%)	0.0004	12.762
Participants used custom-made MG at high school	13 (29.5%)	31 (70.5%)		

**Table 7:** Effect of the mandatory rule at high school on adult wearing rates.

	No MG Use in 2018	MG Use in 2018	*p*-Value	Chi-Square Score
Applied mandatory rule at high school	7 (24.1%)	22 (75.9%)	0.0643	3.4221
No applied mandatory rule at high school	25 (44.6%)	31 (55.4%)		
